# Paradoxical lesions, plasticity and active inference

**DOI:** 10.1093/braincomms/fcaa164

**Published:** 2020-10-01

**Authors:** Noor Sajid, Thomas Parr, Andrea Gajardo-Vidal, Cathy J Price, Karl J Friston

**Affiliations:** Wellcome Centre for Human Neuroimaging, University College London, London WC1N 3AR, UK

**Keywords:** paradoxical lesions, structure–function relationship, plasticity, learning, active inference

## Abstract

Paradoxical lesions are secondary brain lesions that ameliorate functional deficits caused by the initial insult. This effect has been explained in several ways; particularly by the reduction of functional inhibition, or by increases in the excitatory-to-inhibitory synaptic balance within perilesional tissue. In this article, we simulate how and when a modification of the excitatory–inhibitory balance triggers the reversal of a functional deficit caused by a primary lesion. For this, we introduce *in-silico* lesions to an active inference model of auditory word repetition. The first *in-silico* lesion simulated damage to the extrinsic (between regions) connectivity causing a functional deficit that did not fully resolve over 100 trials of a word repetition task. The second lesion was implemented in the intrinsic (within region) connectivity, compromising the model’s ability to rebalance excitatory–inhibitory connections during learning. We found that when the second lesion was mild, there was an increase in experience-dependent plasticity that enhanced performance relative to a single lesion. This paradoxical lesion effect disappeared when the second lesion was more severe because plasticity-related changes were disproportionately amplified in the intrinsic connectivity, relative to lesioned extrinsic connections. Finally, this framework was used to predict the physiological correlates of paradoxical lesions. This formal approach provides new insights into the computational and neurophysiological mechanisms that allow some patients to recover after large or multiple lesions.

## Teaser

Paradoxical lesions are secondary brain lesions that ameliorate functional deficits caused by an initial insult. Using active inference, Sajid *et al.* simulate how and when a modification of the excitatory–inhibitory balance reverses this functional deficit. This provides new (theoretical) insights into one way that patients could recover after secondary lesions.

## Graphical Abstract

**Graphical Abstract:**
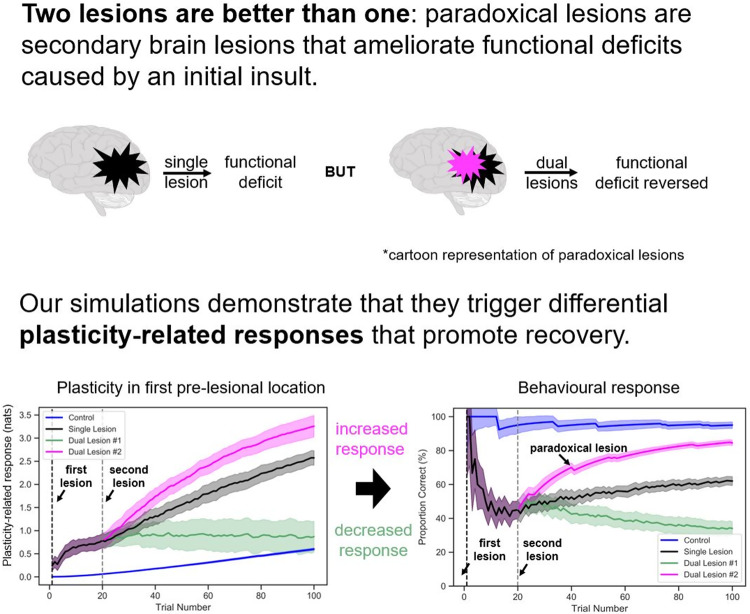

## Introduction

Functional recovery after brain damage is a complex process; influenced by how the initial insult disrupts connectivity amongst intact regions (Nudo, 2006, 2013). Generally, an additional lesion—to a dysfunctional brain—further complicates recovery and can confound functional deficits. However, in some rare instances, an additional (paradoxical) lesion may reverse a cognitive deficit (Kapur, 1996; Kapur *et al.*, 2013). In this paper, we simulate how a paradoxical lesion can facilitate neuroplasticity and help to restore a previously lost function.

Paradoxical lesions were first demonstrated in cats (Sprague and Meikle, 1965; Sprague, 1966*a*, *b*). These seminal studies showed that visual attentional deficits—caused by an initial cortical lesion—were reversed by a secondary lesion in the superior colliculus. These paradoxical effects have been replicated independent of lesion order (Sherman, 1974) and, lesion location in cats (Lomber and Payne, 1996; Payne *et al.*, 1996; Rushmore *et al.*, 2006), or rats (Kirvel *et al.*, 1974; Corwin and Vargo, 1993). Conversely, examples of paradoxical lesions in humans, although rare, have also been reported (Pöppel and Richards, 1974; Vuilleumier *et al.*, 1996; Constantino and Louis, 2003; Weddell, 2004; Mathews *et al.*, 2008; Jha and Brown, 2011). For example (Vuilleumier *et al.*, 1996) revealed that a secondary lesion in the left frontal eye field region reversed left-sided visual neglect caused by right parietal damage.

Two potential explanations of paradoxical lesions have previously been considered (Kapur, 1996; Vuilleumier *et al.*, 1996; Zeiler *et al.*, 2016; Toba *et al.*, 2020; Valero-Cabré *et al.*, 2020). The first is the reduction of functional inhibition (i.e. disinhibition) that might occur if the second lesion damaged inhibitory connections. The other is a restoration of normal interactions between preserved regions that results when the second lesion triggers a critical period of experience-dependent plasticity in perilesional tissue that affects excitatory-to-inhibitory balance, i.e. postsynaptic excitability or cortical gain (Geisler and Albrecht, 1992; Abbott *et al.*, 1997; Pi *et al.*, 2013; Kanai et al., 2015; Mongillo *et al.*, 2018). There is great interest in these neuroplastic changes because they are reminiscent of the critical period in brain development (Schneider *et al.*, 2019) and offer a potential target for interventions to promote and facilitate recovery after brain damage (Starkstein and Robinson, 1997). Multiple factors may alter excitatory–inhibitory balance in perilesional tissue and enable repair (Bansal *et al.*, 2019) including changes in the uptake of neurotransmitters such as glutamate and serotonin and the release of growth factors that promote axonal sprouting in the first 2 or 3 weeks after damage.

To better understand the underlying causal mechanisms, Hilgetag and colleagues (Hilgetag *et al.*, 1999; Hilgetag, 2000) simulated *in-silico* lesions that reversed deficit in a system of coupled ordinary differential equations, with linear connections. Their results showed that functional recovery can result from a re-organization of neuronal connectivity between competing brain regions. We build upon this work to demonstrate how heightened plasticity, due to the modification of the excitatory–inhibitory balance, triggers the reversal of a functional deficit caused by a previous lesion.

For this purpose, we introduce *in-silico* lesions to computational models that produce, and allow for reversal of, a functional deficit using a word repetition task (i.e. hear a word and repeat it back). Our model (and accompanying simulations) should be considered a vehicle to illustrate mechanisms underlying paradoxical lesions—rather than an explanation of how paradoxical lesions have been demonstrated empirically. We modelled the word repetition task using active inference (Friston *et al.*, 2017*a*, *b*). Central to this theory are prior beliefs, about their environment, that patients would have to hold to render their behaviour appropriate (i.e. Bayes optimal) when maximizing model evidence or minimizing free energy. In other words, we move from asking why behaviour appears pathological (i.e. suboptimal) and instead ask ‘what would we have to believe for this behaviour to appear optimal?’ This allows us to characterize patients with brain damage as operating under ideal Bayesian assumptions but with a poor (i.e. lesioned) model of their sensory milieu (Schwartenbeck and Friston, 2016; Parr *et al.*, 2018). Conveniently, active inference also provides a principled way of modelling and measuring (plastic) changes in the synaptic connectivity (Friston *et al.*, 2017*a*).

Our model (and accompanying simulations) should be considered a vehicle to illustrate the mechanisms that may underlie empirically demonstrated paradoxical lesions—and to motivate future studies of whether and how these mechanisms disclose pathways to recovery after neurological damage. For example, we have shown (computationally) how a secondary lesion to the excitatory-to-inhibitory balance in perilesional tissue could trigger a critical period of experience-dependent plasticity. Understanding the neurobiological nature of these mechanisms could lead to novel ways to facilitate the recovery process.

In what follows, we present simulations of paradoxical lesions, with accompanying physiological predictions from the same model, and conclude with a brief discussion of the implications of this kind of *in silico* neuropsychology. In brief, we will see that the simulations endorse the hypotheses (Kapur, 1996; Vuilleumier *et al.*, 1996; Hilgetag *et al.*, 1999; Hilgetag, 2000; Zeiler *et al.*, 2016; Bansal *et al.*, 2019; Valero-Cabré *et al.*, 2020) concerning synaptic disinhibition and plasticity as key mechanisms that underwrite paradoxical lesions. In this sense, the following simulations provide proof of principle that these mechanisms can explain the phenomenology of paradoxical lesions and, furthermore, these mechanisms emerge naturally from a Bayes-optimal response to brain injury.

## Materials and methods

Our aim was to illustrate how secondary lesions to intrinsic connections (within the cortical grey matter) could reverse the functional deficits caused by the initial insult to extrinsic connections (between cortical hierarchies). For this purpose, we used a word repetition task in which the subject repeats a heard word on each trial (Ueno *et al.*, 2011; Moritz-Gasser and Duffau, 2013; Nozari and Dell, 2013; Hope *et al.*, 2014). If the subject repeats the word correctly, they are given a positive evaluation (and negative otherwise). The word repetition task was stimulated using a (Markov decision process) generative model of discrete outcomes (Friston *et al.*, 2017*a*; Sajid *et al.*, 2019; Da Costa *et al.*, 2020), previously introduced in (Sajid *et al.*, 2020). The model considers a left-lateralized neuronal circuitry involved in word repetition, but this can be extended—via additional state factors or hierarchies—to include the right hemisphere. For the interested reader, the Supplementary material provides a detailed description of the generative model (S.1, S.4), accompanying belief updates (S.2) and the learning process (S.3). The generative model—on which these update equations are based—is very general. It can be applied in most settings, where outcomes and their causes can be expressed in terms of distinct (i.e. discrete) states. In addition, the probability distributions that instantiate belief updating were based on an empirical understanding of how subjects respond in a word repetition paradigm. Specifically, belief updating is based on a generative model of how stimuli are produced during an experiment. We assume that real subjects adopt similar generative models when performing this task.

We employ a standard message passing scheme and requisite computational neuroanatomy—defined by the generative model—based on nodes (e.g. neuronal populations) and edges (e.g. neuronal connections) along which messages (e.g. action potentials) are passed (Friston *et al.*, 2017*a*; Parr and Friston, 2018). There are certain aspects of this message passing that can be mapped onto the functional anatomy in the brain e.g. components involved in policy selection ( Friston *et al*., 2014,  2017*a*) or hypothesis-driven assignment of states and outcomes to particular neuronal populations in particular cortical and subcortical structures or, indeed, within the cortical lamina of canonical microcircuits. See Friston *et al.* (2017*b*) for further details and references.

We introduced *in-silico* lesions to the generative model by systematically removing two types of connections that encode different kinds of model parameters: **A** and **B**. These structural (i.e. synaptic) changes have consequences for how the belief-updating unfolds. Here, the parameter matrix **A**—mapping outcomes given their causes—couples adjacent levels of a deep or hierarchical model and can be associated with extrinsic (between region) connectivity. The matrix **A** parameterizes the likelihood, i.e. given the observations about the current verbal cue presented, what is the most likely target word. These structural assumptions mean that we can regard lesions to the **A** matrix as reproducing the kind of disconnections that follow from destruction of white matter tracts; in the sense of Geschwind (Catani and Ffytche, 2005). However, the relationship between a functional and a structural disconnection may be more nuanced. Lesions of extrinsic connections could refer to any pathology of projection neurons, including both axonal (white matter) lesions and or synaptopathy; e.g. Moser and Starr (2016). Conversely, the parameter matrix **B** can be regarded as the intrinsic (within region) connectivity because the transitions are local to a given cortical hierarchy. The matrix **B** parameterizes prior beliefs about state transitions, e.g. given the recent past, what word do I expect to repeat in the present before observing myself repeat it.

A crucial hyperparameter in simulating *in-silico* lesions is precision,ω—which scores confidence in beliefs. Here, precision is the inverse uncertainty over the probabilities in **A** (sensory precision—$\omega_{A}$**)** and **B** (state precision—$\omega_{B}$). For example, if **A** is extremely precise ($\omega_{A}=1$) then the model can be confident that a particular verbal cue (outcome) will be generated reliably by the appropriate target word (cause). In contrast, an extremely imprecise distribution ($\omega_{A}=0.5$) implies an ambiguous relationship between causes and outcomes—and observations do little to resolve uncertainty about their causes. Thus, precision over **A** corresponds to the confidence with which the model can infer a cause from observations and precision over **B** corresponds to confidence with which the model can predict the present from the past (i.e. infer state transitions).

In what follows, plasticity or experience-dependent learning was implemented by accumulating evidence (i.e. pseudo-counts) under the assumption that the parameters of the likelihood and prior transition matrices parameterized a multinomial probability distribution over outcomes and states (S.3). Here, a pseudo-count is an amount added to the number of observed state-outcome or state transition pairs to update the expected probability. This is like remembering the number of times an event has taken place to infer the probability of its recurrence. This follows standard schemes in active inference in which parameters are updated to minimize variational free energy (or maximize and evidence lower bound). Neurobiologically, this corresponds to associative plasticity of a Hebbian sort—see Friston *et al.* (2016) for details.

Plasticity (re-learning) was quantified using Kullback–Leibler (KL)-divergence from the prior to the posterior i.e. a measurement of how the (posterior) probability distribution is different from the (prior) reference probability distribution. Technically, the KL-divergence is between the Dirichlet distributions of our model parameters: the prior is the distribution at the first trial and the posterior is the distribution after each trial following the accumulation of evidence in the form of Dirichlet parameters; c.f., pseudo-counts. These differential learning updates assume that the quality of the observed data (e.g. feedback received by the model) is consistent across precision changes in model parameters, but differences arise due to changes in prior beliefs over model parameters. Intuitively, one should expect precise priors to reveal low KL-divergence, i.e. negligible changes from prior to the posterior distribution, because the cause of the data was predicted confidently—and there is little to learn from the observations. Conversely, imprecise priors are expected to evince greater experience-dependent plasticity and a higher KL-divergence (i.e. changes from prior to the posterior) because the cause of the data was not confidently established prior to the observations and there is therefore more to learn from the observations.

### Data availability

The data presented below has been simulated using generic belief updating and can be implemented using standard routines (here spm_MDP_VB_X.m). These routines are available as MATLAB code in the SPM academic software: http://www.fil.ion.ucl.ac.uk/spm/software/. In addition, the code required to reproduce the simulations and figures has been included in the following GitHub repository: https://github.com/ucbtns/paradoxicalesions.

## Results

### Control model

**Figure 1:**
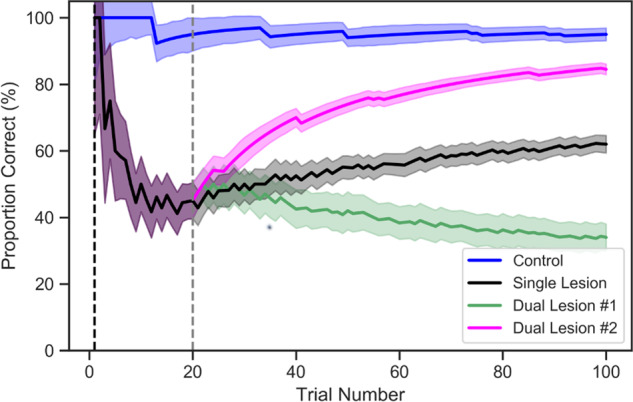
**Proportion correct.** The line plot shows the stimulated (mean) proportion of correct responses for each model across the 100 trials, with 95% confidence interval. The *x*-axis is the trial number and the *y*-axis is the correct number of responses (%). Blue line reports the control model, black line reports the model with single lesion, green line reports the model with dual lesion no. 1 (severe dual lesion) and magenta line reports the model with dual lesion no. 2 (mild dual lesion). The vertical black dashed line represents when the first lesion was introduced (1st trial), and the vertical grey dashed line represents when the second lesion was introduced (20th trial) to the model.

To measure the effect of primary and secondary lesions, we simulated a control model without any lesion. This control model was simulated across 100 trials, for 50 different configurations of the task (based on random initialization seeds). This model had (on average) 95% correct responses, after 100 trials. Figure 1 shows the performance trajectory—as measured by proportion of correct responses (blue line). Here, the appearance of performance degradation is reflective of (on average) 5 incorrect responses across the 100 trials and is an attribute of imprecise action selection (i.e. 4). The precision of action selection is a model hyperparameter that determines how confidently actions are selected during the course of the trial (Schwartenbeck *et al.*, 2015) and is kept consistent across the remaining simulations.

**Figure 2:**
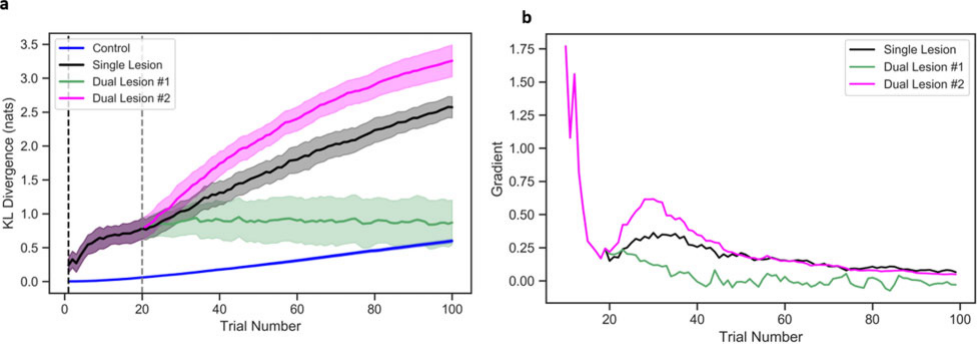
**Measuring plasticity in extrinsic (likelihood) connections **A**— the first lesion location.** (**A**) Plots plasticity in the first lesion location, for each model across 100 trials, with 95% confidence intervals and (**B**) shows the gradients (rate of change) for the plasticity-related changes for the lesioned models over 10 trials to ensure smoothing out of any noise. (**A**) The *x*-axis is the trial number and the *y*-axis represents the KL-divergence (measured in nats) between initial and current distribution. Blue line reports the control model, black line reports the model with single lesion, green line reports dual lesion model no. 1 (with the severe dual lesion), magenta line reports dual lesion model no. 2 (with the mild dual lesion). The vertical black dashed line indicates when the first lesion was introduced (1st trial), and the vertical grey dashed line indicates when the second lesion was introduced (20th trial). (**B**) The *x*-axis is the trial number and the *y*-axis represents the gradient for all lesioned models.

**Figure 3:**
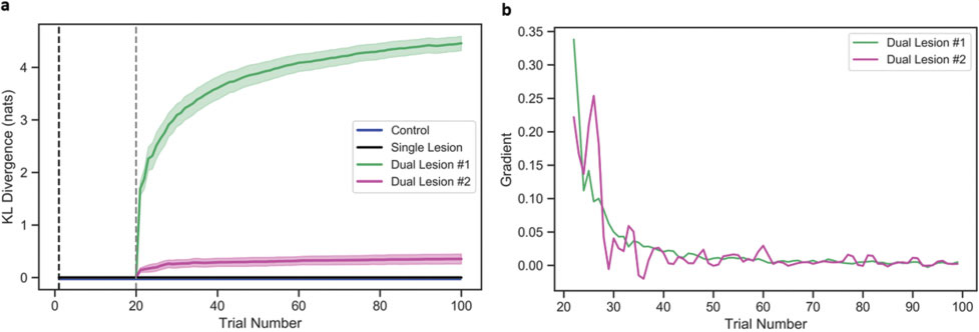
**Measuring plasticity in intrinsic (prior transition) connections B—the second lesion location.** This figure uses the same format as previous figure: (**A**) plots plasticity in the second lesion location, for each model across 100 trials, with 95% confidence intervals and (**B**) shows the gradients (rate of change) for the plasticity-related changes for the lesioned models. (A) The *x*-axis is the trial number and the *y*-axis represents the KL-divergence (measured in nats) between initial and current distribution. (**B**) The x-axis is the trial number and the y-axis represents the gradient for the dual lesioned models.

We also measured the changes in plasticity—using the KL-divergence—for model parameters of interest (Figs 2 and 3—blue line). The negligible change in natural units reflects the model parameterization, which was near optimal: i.e. little to no learning was required.

### Single lesion model

The first lesion was to the parameter matrix **A** (i.e. extrinsic connections): the strength of the most plausible connections was reduced relative to the strength of implausible connections by lowering the precision hyperparameter,$\omega_{A}$, from 1 (as in the control model) to 0.8. This partial disconnection decreases the posterior confidence over the causes of what the model hears and therefore impedes belief updating. We introduced this lesion at the first trial. The ensuing primary lesion model was simulated across 100 trials, for 50 different iterations (using the same random initialization seeds as the control model). By using the same initialization seeds we test for specific counterfactuals, i.e. had it not been for the lesion, the control and this model would have performed in exactly the same way.

The effect of the first lesion is determined by the model’s capacity to update beliefs, which triggers experience-dependent plasticity and changes in KL-divergence. Over time this effect is inevitably diminished as the model becomes confident about particular causes of data (even if they maybe wrong).

This model attained (on average) 62% correct responses, after 100 trials. Figure 1 shows the performance trajectory—as measured by proportion of correct responses (black line). We observed a sharp initial drop in performance that plateaued after the 40th trial. Figure 2 shows the plasticity-related changes for the parameter matrix **A** after the first lesion, i.e. the changes in KL-divergence (Fig. 2A—black line). Initially, there was a heightened period of plasticity: the rate of plasticity-related change (Fig. 2B—black line gradient) was 1.75–1.25 during the first 5 trials. However, there was a slowing of changes after the 20th trial, as the gradient dropped to below 0.2.

### Dual lesion models

The second lesion was to the parameter matrix **B** (i.e. intrinsic connections): the strength of plausible connections was reduced relative to the implausible connections by lowering the precision hyperparameter, $\omega_{B}$ from 1 (in the control and single lesion models) to 0.7 (severe lesion) or 0.9 (mild lesion). These lesions correspond to reduced confidence with which the model can predict the present from the past, relative to control and single lesion models. Anatomically, they imply a disruption to the (intrinsic) recurrent excitatory self-connections that we have associated with transition probabilities; slightly interrupted for mild and substantially interrupted for severe lesions. Heuristically, these can be thought of as different levels of attenuation of the gain of a post-synaptic neuron’s response to a presynaptic afferent. Computationally, slightly interrupted connections (i.e. mild lesions) can still maintain appropriate beliefs over time, in contrast to substantially interrupted synaptic connectivity (i.e. severe lesion). Consequently, experience-dependent plasticity is substantially reduced with severe lesions because beliefs cannot be updated over time—in response to precise fluctuations in pre- and post-synaptic activity (see the belief update equations in the Supplementary material).

We introduced these **B** lesions to two lesion models at the 20th trial. Both lesion models had previously experienced the primary **A** lesion, at the first trial. The effect of the first lesion is negligible at the point of the second lesion i.e. changes in KL-divergence are <0.5 nats (Fig. 2). Similar effects would be expected in late stages of recovery after neurological damage.

The lesion models were simulated across 100 trials, for 50 different iterations (using the same random initialization seeds as the control model). As before, the same seeds imply that with no dual lesions, the control and lesion models would have identical performance. Model no. 1 (severe secondary lesion) had (on average) 34% correct responses, after 100 trials. This simulation had an initial performance drop, as measured by proportion of correct responses, after the second lesion, with performance stabilizing after the 80th trial (Fig. 1—green line). In contrast, model no. 2 (mild secondary lesion) had (on average) 84.5% correct responses, after 100 trials which *is better than the model with the single lesion*. The performance after the mild second lesion improved rapidly, with plateauing after the 80th trial (Fig. 1—magenta line). The second (milder) lesion is an example of a paradoxical lesion, where the second lesion undoes the functional deficit caused by the initial insult. These performance differences are explained below by (i) observing how the system relearns when the ability to update beliefs is progressively impaired and (ii) simulating how electrophysiological responses in the intrinsic connections differ with each type of lesion.

Plasticity-related changes after secondary lesions to the intrinsic connections were evaluated for the both model parameters (**A** and **B**). The effects on the extrinsic connections (**A**) are illustrated in Fig. 2 and the effects on the intrinsic connections (**B**) are shown in Fig. 3. In model no. 1, the second (severe) lesion impedes plasticity in the extrinsic connections **A**—note the negative rate of change in the green line in Fig. 2B—and augments learning in the intrinsic connections **B** as the system attempts to recover (see green line in Fig. 3B where the rate of change is ∼10% to 30% until the 25th trial). That is, a severe lesion to the parameter matrix **B** intensifies the volatility of state transitions and impairs the model’s ability to repeat the target word. Thus, any additional trials do little in terms of resolving uncertainty about states, due to the ambiguous relationship between state transitions, but cause an overall decline in sensory precision via learning as the model recurrently repeats the wrong target word. This results in maladaptive plasticity—and functional recovery.

In contrast, the second (mild) lesion in model no. 2, augments the plasticity in extrinsic connections **A** i.e. an (average) rate of change (gradient) increase in ∼0.3, compared to the single lesion simulation (magenta versus black line in Fig. 2B) and this is maintained over 100 trials (Fig. 2A magenta versus black line). In the intrinsic connections **B**, however, the mild (model no. 2) secondary lesion produces an initial increase in plasticity (∼0.2), compared to the single lesion with no change, which rapidly drops to 0.0–0.4 (magenta line in Fig. 3B). That is, a mild lesion to parameter matrix **B** only minimally affects the precision over state transitions and facilitates the model’s ability to repeat the target word. Thus, additional trials help resolve the (slight) uncertainty about states, by allowing for more confident beliefs over state transitions, and cause an increase in sensory precision as the model learns to repeat the correct target word. This results in adaptive plastic changes in the model.

### Physiological predictions

In the above simulations, we saw that mild secondary lesions to the (within region) intrinsic connections facilitate recovery from an initial lesion to the extrinsic connections. We now investigate how the simulated electrophysiology differs for the different types of lesions investigated. The simulated local field potential responses are based on the belief updating described in the Supplementary material. More concretely, the form of the (variational) message passing mandated by active inference allows us to associate variables with idealized electrophysiological recordings (Friston *et al.*, 2017*a*; Parr *et al*., 2020). Here, we plot local field potentials, after bandpass filtering between 4 and 32 Hz (Friston *et al.*, 2017*a*). This is calculated from membrane depolarization (i.e. post-synaptic potential) gradients computed using the inputs from other neurons.

**Figure 4:**
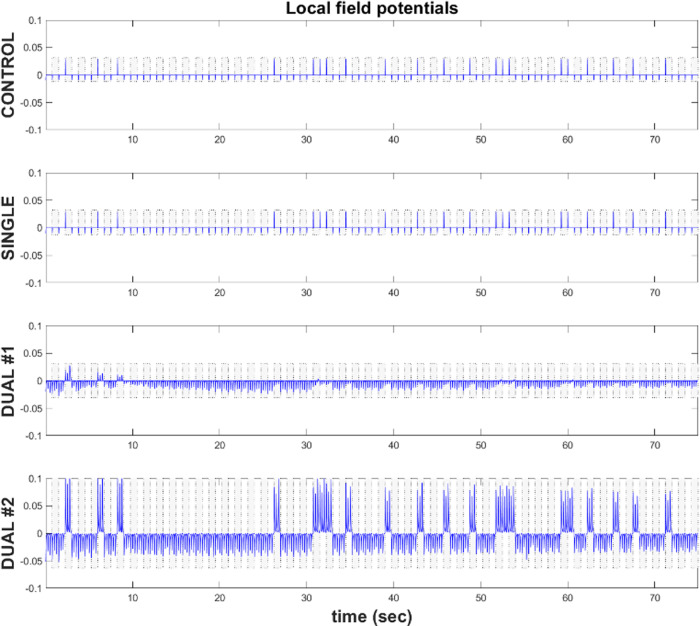
**Simulated local field potentials.** These plots show the simulated local field potentials for each model for a target word across the 100 trials (*x*-axis). These are plotted after bandpass filtering between 4 and 32 Hz (Friston *et al.*, 2017*a*). This is calculated from membrane depolarization (i.e. post-synaptic potential) gradients computed using the inputs from other neurons. The blue shows the trajectory of evoked responses over arbitrary units (*y*-axis), where positive indicates excitatory responses and negative indicates inhibitory responses. The top row presents the control model, the second row shows the single lesion model and the last two rows show the dual lesion models. Each plot represents a single instantiation of the simulated models.

Figure 4 shows these simulated local field potentials for a particular neuronal ensemble at the second lesion location (in the intrinsic connections). Both the control and single lesion simulations (that preserve intrinsic connections) exhibit similar, balanced evoked responses for the duration of the 100 trials. In contrast, the two dual lesion models exhibit a distinct change in evoked responses after the second lesion. Both models exhibit an increase in total inhibitory potential, which is greater with mild (model no. 2) than with severe (model no. 1) lesions. However, while model no. 1 (severe dual lesion) shows an attenuation of excitatory evoked response after the first few trials, model no. 2 (with mild dual lesions) shows a marked increase in excitatory synaptic potential, across trials. The simulated local field potentials therefore illustrate how the mild secondary lesions rebalance the inhibitory synaptic potentials seen with the severe secondary lesions. This results in accentuated excitatory and inhibitory responses compared to control and single lesion model. It is these enhanced responses that may facilitate learning following a mild secondary lesion to the intrinsic connectivity compared to a single lesion to the extrinsic connectivity.

## Discussion

The brain is a dynamic system, where specific (steady) states are determined from complex interactions among neuronal ensembles (Sporns *et al.*, 2004; Parr *et al.*, 2020). Damage to the brain will disrupt these interactions (Kinsbourne, 1970; Alstott *et al.*, 2009). Under some circumstances, the introduction of additional lesions to an already dysfunctional brain may lead to changes that partly or wholly rectify the imbalance by creating or amplifying new kinds of excitatory and inhibitory interactions. In this article, we demonstrate that the severity of the secondary lesion to intrinsic connections can trigger qualitatively different responses; paradoxical or otherwise. Our electrophysiological simulations illustrate that learning differences can manifest as changes to the excitatory–inhibitory balance that underwrite evoked neuronal responses.

Our results highlight a complementary interpretation of the narrative surrounding excitatory–inhibitory balance, in terms of Bayesian inference. This is because we distinguish between two types of parameters—mediating prior beliefs and likelihoods. The intrinsic (**B**) parameters can be regarded as encoding prior beliefs (given the recent past, what is expected in the present before observing it), while the extrinsic (**A**) parameters encode likelihoods (given the states of the world, what is expected to be seen or heard). Precise priors preclude large updates from prior to posterior (i.e. inhibition), while precise likelihoods promote such updates (i.e. excitation). Following a loss of precision in the likelihood, the belief-updating becomes restricted by the prior which, when attenuated, restores balance.

Following on from (Zeiler *et al.*, 2016), our results revealed a self-limiting period of plasticity that could mediate recovery from a previous insult by amplifying and rebalancing excitatory and inhibitory connections—and thereby improving the overall message passing within (a simulated) cortical hierarchy. It is this type of plasticity that may underlie recovery of function after dual lesions. The neurobiological manifestation of this plasticity corresponds to long-term synaptic plasticity, as previously discussed in Friston *et al.* (2017*a*). At the neuronal level, the persistent synaptic activation—determined by changes in the estimated state—induces plasticity leading to either strengthening or weakening of the connections between synaptically connected neurons (i.e. outcomes and states or state transitions). Neurobiological, this can be read as long-term potentiation (Bliss and Lømo, 1973; Malenka and Nicoll, 1999); namely, a persistent strengthening of connections (states-outcomes) based on stimuli (outcomes) or long-term depression (Lynch *et al.*, 1977), which entails a long-lasting decrease in synaptic strength. However, in our model we consider neuronal populations and synaptic plasticity is modelled at an aggregated level.

When the secondary lesion is severe, however, heightened plasticity can be maladaptive—as the lesioned area disproportionally tries to recover lost connectivity (model no. 1 with severe dual lesions). Our simulations therefore highlight a specific type of inhibitory–excitatory balance: increased inhibitory and excitatory synaptic potential—that promotes plasticity. This forms a testable hypothesis for future work: can introducing this particular type of modification to inhibitory–excitatory balance in perilesional regions—via pharmacological modulation or deep brain stimulation (Bestmann, 2015; Little and Bestmann, 2015)—reverse functional deficits? Our results suggest that targeted rehabilitative therapy may only engender functional improvements if it (i) induces wide-spread plasticity in the neural network and (ii) is delivered during the post-infarction sensitive period. In a sense, such therapies tend to target the attenuated likelihood, focusing on re-learning of this excitatory input. There is potential to complement this with concurrent ‘unlearning’ of priors that have become maladaptive.

The main limitation of this work stems from the simplicity of the generative model used and the type of *in-silico* lesions. In most instances, patient data suggests multiple areas lesioned in a single instance of brain damage. However, this model provides new (theoretical) insights into one way that patients could recover after secondary lesions. Specifically, we have shown that functional facilitation, after a secondary lesion to the intrinsic connections, can result from changes in the inhibitory–excitatory balances that promote appropriate levels of plasticity in the overall system. In this work, the results are implementation-agnostic i.e. practically, inhibitory–excitatory changes could have been mediated by transcallosal commissural projections linking homotopic regions (Sprague, 1966*a*; Kapur, 1996) or intra-hemispheric inhibitory–excitatory updates. Future work is needed to distinguish between the two types of inhibitory–excitatory interactions (inter and intra) after an *in-silico* lesion. This would involve equipping the current generative model with additional state factors that represent homologue regions. This would allow the inclusion of other types of damage (e.g. to inhibitory extrinsic connections) that might also facilitate recovery.

In this work, we have focused on discrete definitions of mild and severe lesions; as parameterized by the precision hyperparameter. This allowed us to demonstrate that a secondary mild lesion can trigger paradoxical responses, relative to a severe one. However, for future work, a sufficiently large space of lesion sub-types might need to be considered by adjusting the precision hyperparameter on a more fine-grained scale. This would allow for a more quantitative definition of mild, severe and perhaps other lesions.

Lastly, an interesting avenue of future research would be to focus on whether and how non-invasive neurostimulation techniques such as direct current stimulation, transcranial magnetic stimulation and/or focused ultrasound can induce a paradoxical response (without the need for a second lesion).

## Conclusions

In this paper, we used an active inference model (Friston *et al.*, 2017*a*) to ask how certain secondary brain lesions can reverse the functional deficits caused by an initial insult to the extrinsic connections. By introducing severe and mild secondary lesions to the intrinsic connections, we show that plasticity-related changes and relearning were increased when the second lesion was mild. In contrast, a severe secondary lesion resulted in maladaptive plasticity which impeded relearning. The same model was also used to make physiological predictions, by appealing to Bayesian belief updating schemes used in active inference. The simulated local field potentials suggest that paradoxical functional facilitation is a result of a specific form of inhibitory–excitatory rebalancing: increased inhibitory and excitatory synaptic potentials to evince an apparent increase in cortical excitability. In contrast, non-paradoxical lesions reduced the amplitude of evoked responses. These quantitative predictions indicate how this framework could be used to investigate the neurophysiology of paradoxical lesions.

In summary, the above simulations fully endorse the hypotheses concerning synaptic disinhibition and plasticity as key mechanisms that underwrite paradoxical lesions (Kapur, 1996; Vuilleumier *et al.*, 1996; Hilgetag *et al.*, 1999; Hilgetag, 2000; Zeiler *et al.*, 2016; Bansal *et al.*, 2019; Valero-Cabré *et al.*, 2020). Not only do the simulations offer proofs of principle that these mechanisms can explain the phenomenology of paradoxical lesions, the implicit mechanisms emerge directly from a Bayes-optimal response to brain injury.

## Funding

This work was funded by Medical Research Council (MR/S502522/1 to N.S.; MR/M023672/1 to C.J.P.), Wellcome Trust (Ref: 203147/Z/16/Z and 205103/Z/16/Z to C.J.P. and K.J.F.).

## Supplementary material

Supplementary material is available at *Brain Communications* online.

## Competing interests

The authors report no competing interests.

## Supplementary Material

fcaa164_Supplementary_Data

## Abbreviations

A =: likelihood
B =: state transitions
KL =: Kullback–Leibler
