# Supplementary material for: Paradoxical lesions, plasticity and active inference
Source: Brain Commun. 2020 Oct 1;2(2):fcaa164. doi: 10.1093/braincomms/fcaa164 (PMC7750943; doi:10.1093/braincomms/fcaa164)
Supplement: fcaa164_Supplementary_Data [file fcaa164_supplementary_data.pdf]

Supplementary materials

In order to simulate the word repetition task, we used a partially observable Markov decision process for discrete state space and time, as defined under active inference. In what follows, we provide a brief overview of the model and the accompanying optimisation scheme (S.1-S.3) (Friston et al 2017a). We then describe the particular causes i.e., hidden states (Target Word, Repeated Word and Epoch) and outcome modalities (Proprioception, Evaluation and Word) of interest for a word repetition paradigm (S.4). Using this, we simulate the word repetition task presented in this paper.

S.1 Model parameterisation

Active inference rests on:

- A generative model: $P(o,s,\pi,\eta) = P(\pi \mid \eta)P(\eta)\prod_{t=1}^{T} P(o_\tau \mid s_\tau,\eta)P(s_\tau \mid s_{\tau-1},\pi,\eta)$ with model parameters $\eta = \{A, B, \ldots\}$, over outcomes $o \in O$, states $s \in S$, and action sequences (policies) $\pi = (u_1, u_2, ..., u_t)$ where $u \in U$ and u is a particular action.
- An approximate posterior: $Q(x) = Q(s_0 \mid \pi) ... Q(s_T \mid \pi)Q(\pi)Q(\eta)$ over states, policies and parameters $x = \{s_0, ..., s_T, \pi, \eta\}$

The generative model describes transitions between states in the world that generate observed outcomes. Their transitions depend on action, which depends on posterior beliefs about the next state Here actions are part of the generative process in the world and policies are part of the generative model:

$$
\begin{aligned}
&P(o_\tau \mid s_\tau, A) = Cat(\sigma(\omega_A \log A + 0.1)))\\
&P(A \mid a) = Dir(a)\\
&P(s_\tau \mid s_{\tau-1}, \pi, B) = Cat(\sigma(\omega_B \log B_{\pi\tau} + 0.1)))\\
&P(B_{\pi\tau} \mid b_{\pi\tau}) = Dir(b_{\pi\tau})\\
&P(s_0) = Cat(D)\\
&P(\pi \mid \gamma) = \sigma(-\gamma \cdot G)
\end{aligned}
$$

Here, we have divided the parameters into *A*, *B*, D, a, b and γ. Note that, ω_A and ω_B are the precision hyperparameters—over the model hyper priors *a* and *b*—used to perform *in-silico* lesions. In active inference, all the heavy lifting is done by minimising the free energy with respect to expectations about hidden states, policies, and parameters. Variational free energy can be expressed as a function of the approximate posterior:

$$Q(x) = \arg\min_{Q(x)} F \approx P(x \mid o)$$

Using rules of conditional independence and variational inference, we get the following form:

$$
\begin{aligned}
F &= E_Q[\ln Q(x) - \ln P(x,o)]\\
&= E_Q[\ln Q(x) - \ln P(o) - \ln P(x \mid o)]\\
&= E_Q[\ln Q(x) - \ln P(o \mid x) - \ln P(x)]\\
&= D_{KL}[Q(x) \,||\, P(x)] - E_Q[\ln P(o \mid x)]
\end{aligned}
$$

By taking an additional expectation under $P(o_\tau \mid s_\tau)$, we can predict future outcomes given hidden states (expected free energy) where:

$$G(\pi) = \sum_t G(\pi,\tau)$$
$$G(\pi,\tau) = E_{P(o_\tau|s_\tau)Q(s_\tau|\pi)}[\ln Q(s_\tau \mid \pi) - \ln P(s_\tau, o_\tau)]$$

S.2 Belief update equations:

We optimise expectations about hidden states (including policies and precision) through *inference* and optimise model parameters (likelihood, transition states) through *learning* after a series of observations. This learning and inference entails finding the sufficient statistics of posterior beliefs that minimise variational free energy. This is usually achieved using a gradient descent on free energy (under some policy):

$$\varepsilon_\tau^\pi = \ln A \cdot o_\tau + \ln B_{\tau-1}^\pi s_{\tau-1}^\pi + \ln B_\tau^\pi \cdot s_{\tau+1}^\pi - \ln s_\tau^\pi$$
$$\varepsilon^\gamma = (\beta - \beta_\tau) + (\pi - \pi_0)G$$
$$\beta = 1/\gamma$$

where $\beta_t = \beta + (\pi - \pi_0) \cdot G$ encodes posterior beliefs about precision; π represents the policies specifying action sequences and $\pi_0 = \sigma(-\gamma \cdot G)$. Each s_τ^π represents the expectation of a state at a given time (subscript) under a given policy (superscript).

By minimising these state and precision prediction errors (free energy gradients); i.e., $\varepsilon_\tau^\pi = -\partial_s F$ and $\varepsilon^\gamma = \partial_\gamma F$, we recover the posterior expectations that minimise free energy to provide Bayesian estimates of hidden variables.

S.3 Learning:

The generative model formulation, can be extended to include prior beliefs, over these model parameter priors (i.e., hyperpriors), which are learned through Bayesian belief-updating (Friston et al 2017a, Friston et al 2017b). The natural choice for the conjugate prior is a Dirichlet distribution, given that the probability distributions are specified as a categorical distribution. This means that the probability can be represented simply in terms of Dirichlet concentration parameters (Parr 2019). This allows us to represent each outcome-state (for A) and state-state (B) mapping with Dirichlet parameters:

$$P(A \mid a) = Dir(a) \Rightarrow \mathbb{E}_{P(A|a)}\left[\log A_{ij}\right] = \psi(a_{ij}) - \psi\left(\sum_k a_{kj}\right)$$
$$P(B \mid b) = Dir(b) \Rightarrow \mathbb{E}_{P(B\ |b\ )}\left[\log B_{ij}\right] = \psi(b_{ij}) - \psi\left(\sum_k b_{kj}\right)$$

here ψ is the digamma function.

These Dirichlet parameters can be thought of as ‘pseudo-counts’ i.e., as observations are made the model is able to accumulate Dirichlet parameters that best fit the data.

S.4 Generative model of word repetition

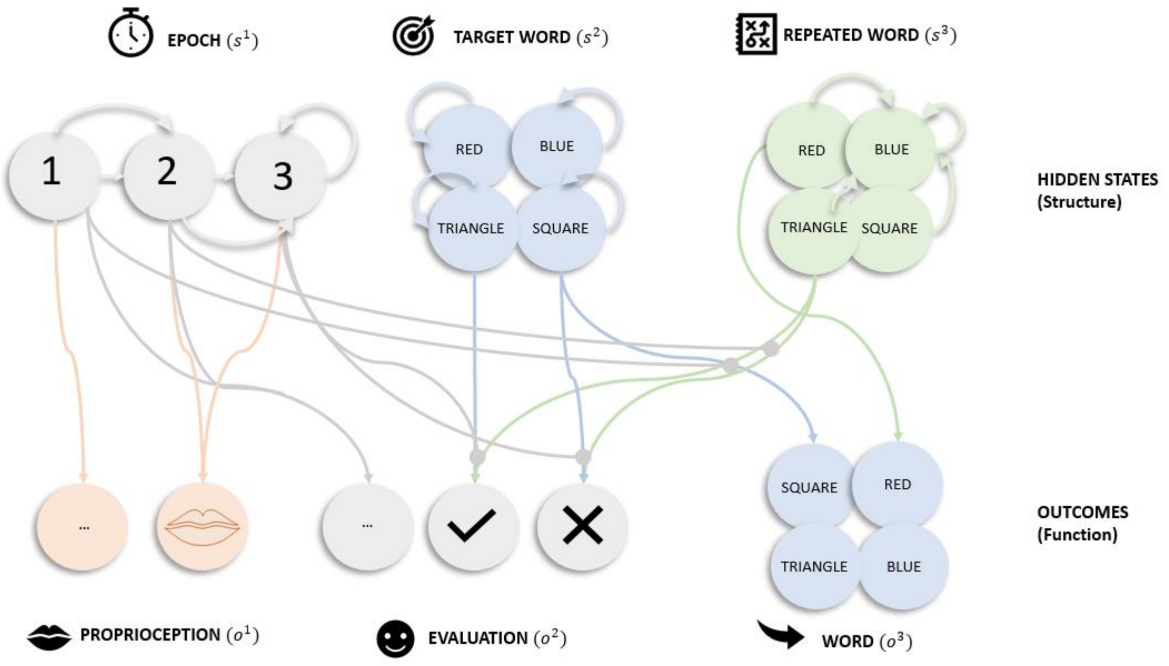

Supplementary Figure 1. Generative model of word repetition

Supplementary Figure 1 is a graphical representation of the generative model for word repetition. There are three (hidden) state factors: Epoch, Target Word, and Repeated Word, and three outcome modalities: Proprioception, Evaluation and Word. The hidden factors had the following levels (i.e., possible alternative states). Epoch (3 levels) indexes the phase of the trial. During the first epoch, the target word is heard. The second epoch involves repeating the word. The third phase elicits a positive evaluation, if the repeated word matches the target word, and a negative evaluation otherwise. The repeated word factor includes the words that model can choose to say (4 levels). The target word factor (4 levels) lists the words the model has to repeat. The lines from states to outcomes represent the likelihood mapping and lines mapping states within a factor represent allowable state transitions. For clarity, we have highlighted likelihoods and transition probabilities that are conserved across the different factors and outcome modalities. For example, the ‘audition’ likelihood mapping target word (square) and audition (square) is shown for Epoch 1, but similar mappings would be applied, when mapping between blue and blue or triangle and triangle. One (out of a total of 4) example transition probability is highlighted for the repeated word, i.e., the transition is always to blue, regardless of previously spoken word (red, triangle, square or blue). This transition represents the choice to say ‘blue’. Similar mappings are applied when choosing to say ‘triangle’, regardless of the previous word. Alternative actions then correspond to alternative choices of transition probability. Note that the lines represent plausible connections (and their absence reflects implausible connections), with the arrow denoting direction. For example, the line mapping hidden state epoch ‘1’ to outcome modality proprioception ‘…’ suggests that ‘…’ is only plausible at epoch ‘1’, but not ‘2’ or ‘3’. Similarly, the line for hidden state target word ‘blue’ to itself reflects that level ‘blue’ can only transition to itself and no other word, throughout the trial.

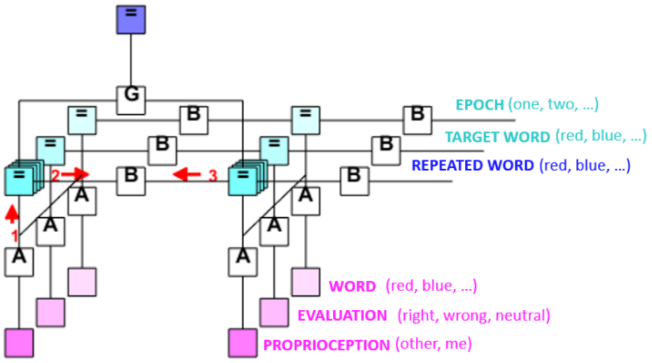

Supplementary Figure 2. Factor graph representation of the generative model

Supplementary Figure 2 is a simplified (normal style) factor graph of word repetition, based upon the likelihood (**A**) and transition (**B**) prior probability matrices (and labels). This is an alternative formulation of the graphical model in Supplementary Figure 1 that makes the message passing clearer. Here, the edges correspond to the sufficient statistics that are passed between the hidden factors denoted by the white boxes. The magenta boxes denote outcomes: audition, evaluation, and proprioception. The cyan boxes denote hidden states: epoch, target word and repeated word. If a hidden state is controllable (i.e., has more than one action dependent probability transition matrix) it is labelled in light blue (and the hidden states are shown as a stack of boxes to indicate they are conditioned on several policies). The key message passing is illustrated with three arrows, corresponding to ascending likelihoods, forward and backward messages (1, 2 and 3, respectively).
